# Intervention for depression among palliative care patients and their families: A study protocol for evaluation of a training program for professional care staff

**DOI:** 10.1186/1472-684X-10-11

**Published:** 2011-06-13

**Authors:** David J Hallford, Marita P McCabe, David Mellor, Tanya E Davison, Denisa L Goldhammer, Kuruvilla George, Shane Storer

**Affiliations:** 1 School of Psychology, Deakin University, 221 Burwood Hwy, Burwood VIC 3125, Australia; 2 Eastern Health, Peter James Centre, Mahoneys Road, Forest Hill VIC 3131, Australia; 3 South West Health, Warrnambool Campus, Ryot St, Warrnambool VIC 3280, Australia

## Abstract

**Background:**

Clinical depression is highly prevalent yet under-detected and under-treated in palliative care settings and is associated with a number of adverse medical and psychological outcomes for patients and their family members. This article presents a study protocol to evaluate a training intervention for non-physician palliative care staff to improve the recognition of depression and provide support for depressed patients and their family members. Details of the hypotheses and expected outcomes, study design, training program development and evaluation measures are described.

**Methods and Design:**

A randomised controlled trial will be implemented across two palliative care services to evaluate the "Training program for professional carers to recognise and manage depression in palliative care settings". Pre-, post- and three-month follow-up data will be collected to assess: the impact of the training on the knowledge, attitudes, self-efficacy and perceived barriers of palliative care staff when working with depression; referral rates for depression; and changes to staff practices. Quantitative and qualitative methods, in the form of self-report questionnaires and interviews with staff and family members, will be used to evaluate the effectiveness of the intervention.

**Discussion:**

This study will determine the effectiveness of an intervention that aims to respond to the urgent need for innovative programs to target depression in the palliative care setting. The expected outcome of this study is the validation of an evidence-based training program to improve staff recognition and appropriate referrals for depression, as well as improve psychosocial support for depressed patients and their family members.

**Trial Registration:**

Australia and New Zealand Clinical Trials Register (ANZCTR): ACTRN12610000183088

## Background

Depression is a significant problem amongst patients receiving palliative care. Studies indicate the prevalence of clinically diagnosable depression in palliative care settings, as defined by the Diagnostic and Statistical Manual of Mental Disorders-IV [1] or International Classification of Diseases-10 [2], is approximately 25 per cent, with up to 50 per cent of patients in this setting reporting high levels of depressive symptomology [3,4]. Factors associated with depression in this population include increased frequency and intensity of physical symptoms, lower general well-being, increased mortality and a hastened desire to die [5-8]. While depression left unaddressed can seriously impact on patients' quality of life, recent evidence supports the efficacy of both pharmacological and psychological treatments in this setting [9,10].

Despite awareness of the high prevalence of depression in this population, rates of detection and treatment are reported to be comparatively low, with only 50 per cent of depressed patients being recognised as depressed and subsequently referred for treatment [11,12]. These low rates of detection strongly indicate the need for improved pathways to care for this vulnerable population. Family members of palliative care patients also represent a high-risk group for psychiatric disorders [13]; yet research indicates they too often do not receive the support needed from professional care services [14,15].

Due to their high level of day-to-day contact with patients, non-physician palliative care staff are in an ideal position to both improve the pathways to care and provide support for depressed patients and their family members. In the project described in this paper, non-physician palliative care staff will participate in a depression training program tailored for the palliative care context as a means to improve their knowledge, attitudes and self-efficacy, and reduce perceived barriers, in regards to detection of depression and the provision of care to depressed patients and their family members. To date, no training program of this type has been evaluated and reported in the scientific literature.

The aim of this paper is to describe the hypotheses of this study, the study design that will be implemented, the development and content of the intervention, and the method of evaluating its efficacy and outcomes.

### Hypotheses and expected outcomes

It is hypothesised that palliative care staff who undertake the depression training program will report higher post-training levels of knowledge, attitudes and self-efficacy and lower perceived barriers in relation to identifying and working with depressed patients compared to pre-training levels and a wait-list control who receive no training. It is also hypothesised that, based on the high prevalence but low detection rates of depression in this context, the number of referrals for depressive symptoms will increase relative to pre-training. The expected outcome of this study is a validated evidence-based program that will assist staff members in recognising depression, increasing appropriate referrals, and improving the care provided for depressed palliative care patients and their family members.

## Methods and design

### Intervention design

The study will constitute a randomised controlled trial, implementing a between-subjects repeated measures design to compare intervention and control conditions over four key areas at three time points. The timeline is outlined in Table 1.

**Table 1:** Evaluation timeline for the Depression Training Program

Evaluation measures for assessing outcomes of the "Training program for professional carers to recognise and manage depression in palliative care settings"	Pre-training	Post-training	3 month follow-up
1. Knowledge of depression	✓	✓	✓
2. View of depression	✓	✓	✓
3. Self-efficacy	✓	✓	✓
4. Barriers to working with depression	✓	✓	✓
5. Number of referrals made for depressive symptoms	✓	✓	✓
6. Family member interviews			✓
7. Staff member interviews			✓

### Target population

The target population will be the non-physician professional care staff that comprise palliative care services. Reflective of the multi-disciplinary nature of these services, and the underlying ideology of active holistic care, health professionals in a broad range of roles will be trained. This will also ensure consistent distribution of information amongst staff and that any gains produced by the training are uniform across the range of services and staff roles. These palliative care staff will be recruited from both in-patient and community palliative care settings.

### Participant groups

A total sample size of 60 was calculated, based on a predicted medium effect size and a statistical power level above 0.8 [16]. Two palliative care services will be recruited into the study with 30 staff from both hospital-based and community based settings recruited from each service. Participants at each site will be randomised to either the intervention condition or the wait-list control condition (no intervention apart from the collection of outcome data). This control group will be offered the training following completion of the study. All attempts will be made to ensure that the sample size and the distribution of participants' professional discipline within the palliative care team (e.g. nursing, occupational therapy, etc.) will be even across sites and conditions.

### Intervention Program: "Training program for professional carers to recognise and manage depression in palliative care settings"

The depression training program, titled "Training program for professional carers to recognise and manage depression in palliative care settings", was developed by drawing from the researchers' experience with depression training in the aged care setting [17,18] and subsequently adapting this framework to the palliative care setting. A literature review pertaining to depression and psychosocial care in palliative care settings was conducted, and information relevant to the objectives of the study was extracted. This evidence-based information was then used to inform the content of the training program. In addition to the literature review, a needs analysis was also conducted which involved focus group interviews with managerial palliative care staff, non-managerial palliative care staff, and family members of patients currently receiving palliative care. These interviews were thematically analysed and information relating to staff knowledge, attitudes, self-efficacy and perceived barriers to depression detection and care provision were extracted and used to further inform the development of the training program. The final program consisted of four sessions focussing on the following main topics: Understanding depression, detecting depression, responding to depression and a focus on the patient's family members. These sessions integrated factual information about depression in palliative care settings (such as its effects or treatment approaches to depression), depression-related and mental health service provision issues unique to this setting (such as distinguishing grief from depression or how depression might be masked), validated approaches to the detection of depression in palliative care (such as a two-question screening tool), and context-specific provision of care for depressed patients and their family members (such as psychosocial care strategies). The specific content of the training program is outlined in Table 2.

**Table 2:** Delivery format and content of the "Training program for professional carers to recognise and manage depression in palliative care settings"

Session 1 Understanding Depression	Session 2 Detecting Depression	Session 3 Responding to Depression	Session 4 Family Focus
• diagnostic definitions and prevalence • signs and symptoms of depression useful for detection in the palliative care setting • distinguishing grief and depression • impact on physical, psychological and social areas at end-of-life • treatments • common misconceptions • distinguishing depression and anxiety	• using a depression monitoring checklist [17,18] * • communication skills • factors related to masked depression in palliative care settings • barriers to detection of depression in palliative care settings	• using a two-question depression screening tool [19,20] ** • responding to depression-related and 'desire to die' statements • support strategies for depressed patients	• psychological distress amongst patient's family members • the family system • support strategies for family members of patients • grief and bereavement • cultural considerations • self-care strategies for staff

*This depression monitoring checklist has been adapted from a similar checklist that has demonstrated success in the aged-care setting in assisting staff to recognise depression **This screening tool specifically targets clinical depression, has been validated against reliable and valid measures of depression, and has demonstrated good psychometric properties in the palliative care setting.

The intervention is designed to be delivered in four 90-minute sessions over the course of four consecutive weeks. This format was chosen so as to allow participants to engage in simple homework tasks between sessions to facilitate the transfer of learned skills to daily practice, for example, trialling methods of detecting depression or implementing support strategies. The outcomes of set homework tasks will be discussed in a group format at the start of sessions two, three and four, so feedback can be given and any questions or issues addressed. Group discussions such as these will be encouraged throughout the program to complement the individual and group activity worksheets used alongside information delivered didactically in a lecture-style format. This dynamic format has been chosen to encourage both the learning of the program content and the sharing of experiences and perspectives amongst palliative care staff in diverse roles.

A presenter's manual and slide presentation has been developed and will be accompanied by a training support kit for participants that includes worksheets, a copy of the slide presentation, and information on resources to provide to depressed patients and family members.

### Evaluation of the training program

#### Palliative care staff

Assessment of the program will be achieved by using evaluation measures completed by participating staff in the intervention and control groups pre- and post-training, as well as at a three-month follow-up time point (refer to Table 1). As measures of the variables targeted by this intervention have not previously been developed specifically for use in the palliative care context, measures validated in other settings were modified for use in this setting. These were as follows:

1. *Knowledge of depression*. This measure was developed by the research team as a means of assessing palliative care staffs' general knowledge about depression. The questionnaire contains 30 items covering knowledge of the signs and symptoms of depression, facts relating to the impact of depression, and common misconceptions about depression. This scale consisted of items from the *Knowledge of Depression Scale *[21], which has demonstrated good psychometric properties with aged care staff, and items derived from a pool of knowledge-based questions created by the researchers that are specifically relevant to the palliative care setting. These items were informed by the relevant empirical literature and the needs analysis that was conducted prior to program development/modification. An example of an item from this scale is, "Depression is often associated with a hastened desire to die". Responses are measured on a four-point Likert scale ranging from 'strongly disagree' to 'strongly agree'.

2. *Views of depression. *This questionnaire consists of 21 items and will be used to measure staff member's attitudes towards depression and the provision of mental health care. It was constructed from items from a modified version of the *Depression Attitude Questionnaire *[22] and items from a pool of attitude-based questions derived using the same process described above. An example of an item from this scale is, "It is important that carers spend time with patients discussing how they are coping psychologically". Responses are measured on a five-point Likert scale ranging from 'strongly disagree' to 'strongly agree'.

*3. Self-efficacy in detecting and managing depression. *This 16-item questionnaire was adapted to the palliative care setting from a scale originally developed to assess the self-efficacy of care staff in working with depression in the aged care sector [17]. An example of an item from this scale is, "In knowing when it might be time to raise concerns about a patient who might be depressed, I feel...". Responses are measured on a four-point Likert scale ranging from 'not at all confident' to 'very confident'.

4. *Barriers to detecting and managing depression. *This 12-item questionnaire will be used to assess staff members' perceived barriers to detecting depression and providing care for depressed patients and their family members. The items from this scale were constructed from a pool of items created by the researchers and based on the barriers to detection and management of depression identified in the literature review and needs analysis. An example of an item from this scale is, "The stigma associated with depression makes it difficult to talk about such issues with patients and family members". Responses are measured on a four-point Likert scale ranging from 'strongly disagree' to 'strongly agree'.

Semi-structured interviews will be conducted at the three month follow-up with groups of care staff who have participated in the training program to obtain their feedback on the program and how it may have impacted on their practices and level of knowledge, views, self-efficacy and perceived barriers towards working with depression among their patients. These interviews will supplement the data collected in the quantitative measures by gathering more in-depth information and providing a forum for staff to advise on aspects of the training program which they found particularly helpful or informative, aspects that may benefit from further refinement, or by providing other information that may not be captured in the measures. The data collected by both the qualitative and quantitative methods will be used to amend and refine the training program for future delivery to palliative care staff.

#### Patients and their family members

Due to the sensitive nature of research in the palliative care setting no data will be collected directly from patients. Outcomes for patients and their family members will be measured via two methods (refer to Table 1). Firstly, rates of referrals made to specialist health services for depression or suspected depression will be collected at pre-training, post-training, and three-month follow-up points from both the intervention and control facilities. This will allow any increase in patient referrals, compared to the control group, as an effect of staff training to be gauged. Secondly, 30 family members of patients receiving palliative care from the service before and after the training is implemented will be interviewed to determine any perceived changes in staff practices as a result of the training, and the associated outcomes on quality of life for those in their care. A semi-structured approach will be used for these interviews to target areas of potential change whilst also allowing for family members to discuss issues around the provision of depression-related care for their relatives and themselves.

### Data analysis

Quantitative and qualitative analysis of the data, and comparison of intervention and control group data, will be used to provide insight into changes in the variables targeted by the interventions, the process of change produced by the targeted intervention, as well as any resulting changes in patient referrals for depression.

The effectiveness of the depression training program will be evaluated using repeated measures multivariate analysis of variance with study condition (intervention or control) as the independent variable and the palliative care staff outcome measures as dependant variables. This type of analysis will facilitate the assessment of changes in staff knowledge, attitudes, self-efficacy and perceived barriers to care for depressed patients and their family members.

All qualitative interviews with staff and family members will be recorded and transcribed. The research team will analyse the interviews to identify and interpret the key themes arising. These themes will be independently identified by members of the research team and cross-checked for inter-rater reliability.

### Consent and Ethics

All participating palliative care staff and patient family members will be provided with a plain language statement and will provide written informed consent to participate in training and/or interviews. Various strategies will be used to maximise the response rate of participating staff members, and participating family members will be offered gift vouchers to compensate for their time. The project has been approved by the Deakin University Human Research Ethics Committee (DU-HREC 2009-194), Eastern Health Research and Ethics Committee (E87/0910) and South West Healthcare Multidisciplinary Ethics Committee (1/2010).

## Discussion

Depression is a highly prevalent psychological disorder in palliative care settings and is associated with a reduced quality of life for patients and substantial costs to the health care system [23,24]. Expert working groups around the world have noted the poor corresponding levels of recognition and treatment of depression in this setting, and have identified the need for the implementation of more effective pathways to treatment and increased provision of care [25,26]. The project described in this article responds to this need by proposing an intervention to address these issues. The proposed training intervention aims to increase palliative care staff members' ability to better understand, recognise and provide care for those suffering from depression which, in turn, will result in an increased quality of life for patients and their family members.

The information gained from this study will shed light on the processes involved in pathways to care for depressed patients as well as contribute to a better understanding of staff factors that relate to the identification and treatment of depression among palliative care patients and their families. More specifically, it will provide an evaluation of the efficacy of this training program and significantly add to the evidence base of effective interventions in this area.

## Competing interests

The authors declare that they have no competing interests.

## Authors' contributions

MPM, DM and TED conceived the project and secured the project funding. They will manage the project. DJH and DLG will work on the project. DH produced the first draft of this paper which has been reviewed by all authors. All authors have read and approved the final manuscript.

## Pre-publication history

The pre-publication history for this paper can be accessed here:

http://www.biomedcentral.com/1472-684X/10/11/prepub
